# Motility of glioblastoma cells is driven by netrin-1 induced gain of stemness

**DOI:** 10.1186/s13046-016-0482-0

**Published:** 2017-01-09

**Authors:** Irene Ylivinkka, Harri Sihto, Olli Tynninen, Yizhou Hu, Aki Laakso, Riku Kivisaari, Pirjo Laakkonen, Jorma Keski-Oja, Marko Hyytiäinen

**Affiliations:** 1 Translational Cancer Biology Research Program, Faculty of Medicine, University of Helsinki, Helsinki, Finland; 2 The Hospital District of Helsinki and Uusimaa, Helsinki, Finland; 3 Department of Pathology, Haartman Institute, University of Helsinki and HUSLAB, Helsinki, Finland; 4 Department of Neurosurgery, Helsinki University Hospital and Clinical Neurosciences, Neurosurgery, University of Helsinki, Helsinki, Finland; 5 Translational Cancer Biology Research Program, Biomedicum, University of Helsinki, B530b2, PL 63 (Haartmaninkatu 8), 00014 Helsinki, Finland

**Keywords:** Netrin, Glioma, Glioblastoma, Glioblastoma stem-like cell, Cancer stem cell, Notch signalling, Cell invasion

## Abstract

**Background:**

Glioblastoma is an untreatable brain cancer. The tumors contain a population of stem-like cells which are highly invasive and resistant to therapies. These cells are the main reason for the lethality of glioblastoma. Extracellular guidance molecule netrin-1 promotes the invasiveness and survival of various cancer cell types. We have previously found that netrin-1 activates Notch signaling, and Notch signaling associates with cell stemness. Therefore, we have here investigated the effects of netrin-1 on glioblastoma pathogenesis and glioblastoma cell stemness.

**Methods:**

Glioma tissue microarrays were stained with immunohistochemistry and the results were used to evaluate the association between netrin-1 and survival of glioma patients. The localization of netrin-1 was analyzed utilizing fresh frozen glioblastoma tissues. The glioma cell invasion was investigated using ex vivo glioma tissue cultures and newly established primary cell cultures in 3D in vitro invasion assays. Intracranial mouse xenograft models were utilized to investigate the effects of netrin-1 on glioblastoma growth and invasion in vivo.

**Results:**

Netrin-1 expression associated with poor patient prognosis in grade II-III gliomas. In addition, its expression correlated with the stem-like cell marker nestin. Netrin-1 overexpression in cultured cells led to increased formation of stem-like cell spheroids. In glioblastoma tumor biopsies netrin-1 localized to hypoxic tumor areas known to be rich in the stem-like cells. In xenograft mouse models netrin-1 expression altered the phenotype of non-invasive glioblastoma cells into diffusively invading and increased the expression of glioma stem-like cell markers. Furthermore, a distinct invasion pattern where netrin-1 positive cells were following the invasive stem-like cells was detected both in mouse models and ex vivo human glioblastoma tissue cultures. Inhibition of netrin-1 signaling targeted especially the stem-like cells and inhibited their infiltrative growth.

**Conclusions:**

Our findings describe netrin-1 as an important regulator of glioblastoma cell stemness and motility. Netrin-1 activates Notch signaling in glioblastoma cells resulting in subsequent gain of stemness and enhanced invasiveness of these cells. Moreover, inhibition of netrin-1 signaling may offer a way to target stem-like cells.

**Electronic supplementary material:**

The online version of this article (doi:10.1186/s13046-016-0482-0) contains supplementary material, which is available to authorized users.

## Background

Glioblastoma (GBM) is the most common primary human brain tumor. Despite the research efforts during recent years this cancer has remained incurable. The median survival time of patients is 15 months after diagnosis. GBMs are extremely heterogenous tumors with high invasive capability. The tumors are rich in cells that possess characteristics of stem cells [15, 35, 36]. These cells, named GBM stem-like cells, show self-renewing capability, can differentiate into different brain tumor cell types and express various neural stem cell markers. GBM stem-like cells are suggested to be the main reason for the lethality of GBMs because of their invasiveness and resistance to radiotherapy [3].

Netrin-1 (NTN1) is a secreted component of the extracellular matrix [31]. Its main function is to guide the developing axons into their correct targets during embryogenesis [30]. Within recent years NTN1 has shown out to be essential for the tumorigenesis of different cancers including neuroblastoma, non-small cell lung cancer, pancreatic adenocarcinoma, metastatic breast cancer and colorectal cancer [9–11, 13, 20, 27]. We and others have observed that NTN1 can enhance the invasive phenotype of GBM cells [33, 41]. Furthermore, we have shown that the increase in in vitro invasiveness is mediated by NTN1 induced activation of Notch signaling [41].

Based on the previous findings we explored the role of NTN1 using human GBM samples and in vivo models. Because Notch signaling is also essential for the maintenance and motility of GBM stem-like cells [12, 17] we analyzed how NTN1 affects these cells. We report here that NTN1 is associated with poor patient prognosis in low grade gliomas. In addition, its expression correlates with the GBM stem-like cell marker nestin. In xenograft models NTN1 expression converted the phenotype of non-invasive GBM cells into diffusively invading and increased the expression of GBM stem-like cell markers. Furthermore, NTN1 and Notch signaling inhibition with recombinant NTN1 derived peptide restricted the GBM stem-like cell infiltrative growth in vivo. These findings provide new information on the regulation of GBM stem-like cells and their motility.

## Methods

### Reagents and antibodies

NTN1 recognizing antibody (CH23002) was obtained from Neuromics. Monoclonal antibody against CD133 (W6B3C1, MACS Miltenyi), polyclonal antibody against nestin (3579, Millipore) and polyclonal antibody against Sox2 (Cell Signaling) were used to recognize the stemness markers of GBM cells. Anti hemaglutinin antibodies from Covance (HA-11) and from Sigma-Aldrich (clone 3 F10) were used to recognize tagged NTN1. The tumor vasculature was stained with mouse CD31 recognizing antibody from R&D Systems. Notch2 extracellular domain (25–255) and intracellular domain (D67C8) recognizing antibodies were obtained from Santa Cruz Biotechnology and Millipore respectively. Antibody recognizing cleaved Notch2 (D1733) was obtained from Immunoway. Intracellular domain of Jagged1 (C-20) recognizing polyclonal antibody was obtained from Santa Cruz Biotechnology. Human specific lamin A/C antibody (Novus Bio) was used to distinguish human tumor cell in mice xenografts. The fluorochrome conjugated Alexa secondary antibodies used in immunofluorescence microscopy were purchased from Invitrogen. The nuclei were visualized with Hoechst 44432 (Invitrogen). Recombinant human EGF, basic FGF and NTN1 proteins were obtained from R&D Systems. Growth factor reduced Matrigel was obtained from Corning.

### Cell-lines and culturing

U87MG (ATCC), U251MG (Health Sciences Research Resources Bank, Japan) and U373MG cells (obtained from Dr. Bengt Westermark, Uppsala) cells were grown in Dulbecco’s modified essential culture medium (DMEM) supplied with 10% heat inactivated fetal calf serum (FCS) (Gibco, USA), 100 IU/ml penicillin, 50 mg/ml streptomycin and 1% L-glutamine. 293FT cells (Invitrogen, USA) were grown in DMEM supplied with 4.5 μg/ml glucose, 10% heat inactivated FCS, 100 IU/ml penicillin, 50 mg/ml streptomycin and 1% L-glutamine. Primary GBM cells, GBM9 and GBM10, were grown in DMEM/F12 cell culture medium (Gibco) supplemented with 100 IU/ml penicillin, 50 mg/ml streptomycin, 20 ng/ml basic fibroblast growth factor (bFGF, R&D Systems), 20 ng/ml epidermal growth factor (EGF, R&D Systems), B27 supplement without vitamin A (Gibco).

### Human tumor tissue biopsies

The use of human tumor tissue and clinical data was approved by the Operative Division of the Ethical Committee of Helsinki University Hospital and The National Supervisory Authority for Welfare and Health. Tumor tissue samples were collected from the archives of Department of Pathology, Helsinki University Central Hospital, Finland and tissue microarrays were constructed from representative tumor areas. Two paraffin embedded tissue microarrays were studied: an array containing 112 low grade glioma samples previously described in [38] and an array consisting of 40 primary GBMs [34]. The tumors were diagnosed and graded according to the WHO 2007 classification [22]. 1p/19q codeletion status was available for 35% of the oligodendroglial tumors. For the rest of the oligodendrocytic tumors the correct classification was ensured with p53 immunohistochemistry. Only samples whose histological type, grade, gender, age at the time of diagnosis, follow-up time until death and successful NTN1 staining were available, were included in analyses.

In addition, we collected fresh GBM tumor biopsies directly from the surgery. The study protocol for obtaining patient samples was approved by the Operative Division of the Ethical Committee of Helsinki University Hospital. All patients gave their written informed consent. The inclusion criteria were age over 18 years, recent magnetic resonance imaging demonstrating a brain tumor with typical GBM appearance, and such tumor location that sufficient, representative tumor biopsy (approximately 1 cm^3^) could be safely obtained during tumor resection. Additional tumor biopsies were collected for routine clinical immunohistopathological analysis. The tissue was washed with DMEM/F12, embedded into OCT compound (Sakura Biotech) in a cryomold and fresh frozen using liquid nitrogen. After freezing the tissueblock was stored a −80 °C. For immunofluorescence staining 7 μm thick sections were cut with Cryotome (Sakura Biotech) and transferred onto microscope slides for staining. The slides were either stained directly or stored in −20 °C.

### Immunohistochemical staining of paraffin embedded tissue

To evaluate the expression of NTN1 using immunohistochemistry, 5 μm thick tumor sections were cut on SuperFrost^+^ slides (Menzel-Gläser). First the sections were deparaffinized in xylene and rehydrated in a decreasing ethanol gradient series. Endogenous peroxidase activity was quenched by incubating the sections in 3% hydrogen peroxide for 30 min. After that, the epitope was retrieved using heat induced epitope retrieval in sodium citrate buffer (10 mmol/L, pH 6.0). Sections were autoclaved 120 °C for 2 min. Next, the NTN1 antibody dilution was administered to the sections and incubated overnight in +4°. Primary NTN1 antibody was detected using rabbit anti chicken secondary antibody followed with ImmPress (Vector laboratories) staining kit used according to manufacturer’s instruction. The bound NTN1 antibody was finally detected with a DAB Peroxidase Substrate Kit (Vector Laboratories) 15 min at room temperature. The slides were counterstained using hematoxylin. Kidney tissue was used as positive control. The NTN1 staining was classified as either positive or negative. Immunohistochemistry protocol to stain nestin is described elsewhere [28].

### Lentiviral gene transduction

Cells stably expressing firefly luciferase and NTN1, or green fluorescent protein and NTN1 were produced with lentiviral transduction as previously described in [41]. Shortly, lentiviruses were produced in 293FT cells by Turbofect (Thermo Fisher Scientific) transfection according to manufacturer’s instructions. Viruses were collected and virus titer determined 48 h after transfection. Target cells were infected with then infected with tittered lentiviruses. For intracranial xenograft experiments U87MG and U373MG cells were transduced with a pLVX-hygro plasmid carrying LUC2 firefly luciferase gene (Promega). Stable LUC2 expressing cell pools were selected with 200 μg/ml hygromycine (Sigma-Aldrich) for 48 h. These cells were further transduced with either pLVX-puro vector, full-length NTN1 fused with Flag or hemagglutinin (HA) tags (NTN1FH) or with NTN1 central fragment (amino acids 282–486 of full-length NTN1) fused with Flag and HA tags (NTN1(II)FH) [41]. The transduced cells were selected by administering 5 μg/ml puromycine (Calbiochem) in their culture medium. The efficiency of the overexpression of LUC2 and NTN1 was evaluated by bioluminescence quantitation and western blotting.

For 3D cell invasion assays U251MG cells stably expressing empty pLVX-Puro vector, NTN1FH or NTN1(II)FH were further lentivirally transduced with pLenti CMV GFP Puro plasmid. The pLenti CMV GFP Puro plasmid was a gift from Eric Campeau (Addgene #17448) [14]. Pool of GFP positive cells were then sorted with fluorescence activated cell sorting using Sony SH800Z Cell Sorter.

### Orthotopic glioblastoma xenografts

All experimental procedures involving mice were authorized by the National Animal Experiment Board. U87MG and U373MG cells expressing either firefly luciferase and NTN1FH or NTN1(II)FH were implanted into the brain of BALB/C NU/NU athymic mice. Five mice per cell-line were used. Intracranial implantation of the cells was performed as previously published [18, 24]. Shortly, cells were trypsinized, counted and suspended into PBS. 150 000 cells in 5 μl were loaded into the Hamilton needle. The mice were anaesthetized using combination of ketamine and xylazine. Skin on mouse scalp was opened with sagittal incision and a hole was drilled into the mouse scull. The hole was positioned 2 mm right and 1 mm anterior to the bregma suture. Using stereotactic device and the Hamilton needle was inserted 3 mm deep into the mouse brain. After 1 min the needle was lifted 0,5 mm. The cells were injected into the brain during 2 min and allowed to stabilize for 1 min. After injection the Hamilton needle was slowly removed, the injection hole on the skull was covered with bone wax and the wound closed with a stitch. The recovery of the mice was enhanced by administering Temgesic (RB Pharmaceuticals) 0,3 μg/mouse on the operation day and on two following days.

The tumor growth was followed with bioluminescence imaging. 3 mg of D-Luciferin (Regis Technologies) in PBS was administered subcutaneously to each mouse and allowed to circulate for 15 min. The mice were anaesthetized with isoflurane and the emitted photons were measured using Perkin-Elmer IVIS 100 imaging system. In addition, the weight of the mice was monitored regularly to evaluate their physical condition. The mice were sacrificed 21 days after U87MG implantations and 52 days after U373MG implantation.

After sacrificing the mice their brain was immediately collected and fixed with 4% paraformaldehyde in phosphate buffered saline (PBS) for 30 min. The tissue was then washed with PBS and incubated with 30% sucrose for 24 h. After that the tissue was embedded into OCT compound (Sakura Bioteh) in a cryomold (Sakura Biotech) and frozen using liquid nitrogen. The frozen tissue blocks were stored in −80 °C. For immunofluorescence staining, 7 μm thick sections were prepared and transferred onto SuperFrost^+^ slides (Menzel-Gläser). Slides were either processed immediately or stored in −20 °C.

### Tissue immunofluorescence staining

Frozen tissue of both mice and human were used for immunofluorescence stainings. Frozen, paraffin fixed mouse tissue sections were melted in room temperature for 5 min, fixed with cold acetone 10 min and washed with PBS. The unfixed, fresh-frozen human tumor biopsies were melted and washed with PBS. The following procedures were same for both tissue types. The unspecific binding of primary antibody was prevented by incubating the sample for 30 min with 0,5% casein in tris buffered saline (TBS) (blocking buffer). Tissue was incubated with primary antibody diluted to blocking buffer at +4 °C overnight. Next, slides were washed with 0,05% Tween in TBS and re-incubated with blocking buffer. Fluorescent labelled secondary antibody and Hoechst for recognizing nuclei were diluted to blocking buffer, administered to the tissue sections and incubated at room temperature for 1 h. Slides were washed with 0,05% Tween in TBS and distilled water. After that slides were mounted with Mowiol mounting medium (Sigma) and covered with cover glass (Menzel). Tissues were analyzed under either Zeiss Axioplan fluorescence microscope, Zeiss LSM 880 Confocal microscope or Zeiss Axio Imager.Z1 upright epifluorescence microscope.

### Ex vivo GBM tissue culture

Fresh GBM tissue biopsies were rinsed with DMEM/F12 and cut into 2 mm X 2 mm pieces. Each tissue piece was then embedded into 50 μl of growth factor reduced, phenol free Matrigel (Corning) and transferred on to a center of a well of 24-well cell culture plate (Thermo-Scientific). The Matrigel was allowed to polymerize for 30 min. DMEM/F12 supplemented with 100 IU/ml penicillin, 50 mg/ml streptomycin, EGF 20 ng/ml (R&D systems), bFGF 20 ng/ml (R&D systems) and vitamin A depleted B27 supplement (Gibco) was administered to the well. The growth of the tumor was assessed under Zeiss Axiovert 200 inverted transmitted light microscope. The tumor tissue was allowed to grow until half of the area of the Matrigel drops were covered by the sprouting tumor cells, typically 7 days. The drops were then transferred into OCT compound (Sakura Biotech) filled cryomolds (Sakura Biotech), frozen and stored similarly as GBM tissue biopsies.

### In vitro cell proliferation assay

Proliferating cells were labelled using Click-IT EdU Imaging Kit (Life Technologies) following the standard protocol of the manufacturer. In short, U87MG or U373MG cells were pulsed with 10 μM of EdU for 60 min prior to fixation. Cells were stained using Click-IT EdU Imaging Kit with AlexaFluor-594 conjugate (Thermo Scientic). The nuclei were visualized with 5 μg/ml of Hoechst 44432 (Invitrogen). The proliferation rate was analyzed using ANIMA software [29]. In short, cells were segmented for nuclear Hoechst signal. Resulting nuclear masks were used to measure the intensities of EdU. The EdU positive cells were selected by k-means clustering algorithm and the proliferation ratio was determined by dividing the total cell number with EdU labelled cell number.

### Neurosphere formation assay

Cells were harvested by trypsinization and washed with serum-free DMEM/F12 medium (Lonza). Cells were pelleted by centrifugating 500 g 5 min and resuspended into DMEM/F12 medium supplied with 50u/ml Penisillin, 50 μg/ml Streptomycine, 20 ng/ml bFGF (R&D Systems), 20 ng/ml EGF (R&D Systems) and B27 (Gibco). 100 cells were seeded per well on a 96-well plate. Cells were incubated for 3 weeks and the number of the formed neurospheres was quantitated. The stemness of neurspheres was ensured by examining their mRNA expression of GBM stem-like cell markers nestin, Sox2 and integrin alpha 6 with quantitative real time PCR. RNA from the cells was collected and real-time PCR was performed as described earlier [41]. The primers for the analysis were obtained from Applied Biosystems. The expression levels were normalized to the expression of glyceraldehyde 3-phosphate dehydrogenase (GAPDH) and compared to wildtype U251MG cells.

### Neurosphere invasion assays

The Boyden chamber invasion assays were performed as previously described in [41]. Shortly, U251MG neurospheres were collected, dissociated and plated on top of Matrigel coated Boyden chamber. Either DMEM/F12 as such or supplemented with 50 ng/ml of recombinant NTN1 (R&D Systems) was added to the lower chamber. Cells were allowed to migrate for 8 h and then fixed, stained and quantitated.

For 3D Matrigel invasion assays U251MG neurospheres were collected and mixed with 50 μl of Matrigel matrix. 5 spheroids per 50 μl of Matrigel was collected. The plug was plated on 24-well plate and allowed to polymerize at +37 °C 30 min. DMEM/F12 culture medium as such or supplemented with 50 ng/ml recombinant NTN1 was added to the wells. The growth of the spheroids were monitored with Cell-IQ imaging system (Chip Man Tehcnologies) and 10x objective. Each spheroid was imaged every 30 min for 24 h. Using Cell-IQ software the growth of the spheroids was analyzed by measuring its area at each imaging timepoint. In addition the images of each spheroids were combined to a movie using 7 frames per second display rate.

### Establishment of primary GBM stem-like cell cultures

The fresh GBM tissue obtained from surgical tumor resection was processed according to previously published protocol [16]. Shortly, the tissue pieces were minced carefully and washed with DMEM/F12 medium supplemented with 100 IU/ml penicillin and 50 mg/ml streptomycin. After washing the medium was replaced with 5 ml Trypsin EDTA (Gibco) and incubated at +37 °C for 30 min. The detachment of cells was ensured by vigorous pipetting up and down every 10 min. The trypsin was inhibited by adding 10 ml of Defined trypsin inhibitor (Gibco). The non-dissociated tissue pieces were allowed to sediment and the medium containing single cells was moved into a new tube. The cells were collected by centrifuging 1000 rpm 5 min. Cells were resuspended into DMEM/F12 supplemented with 100 IU/ml penicillin, 50 mg/ml streptomycin, EGF 20 ng/ml (R&D systems), FGF 20 ng/ml (R&D systems) and vitamin A depleted B27 supplement (Gibco) in a density of 50 000 cells/ml and plated into cell culture flasks. The flask were incubated in cell culture incubator for 7 days for neurospheres to form. The culture medium was changed every second day. The neurospheres were trypsinized and plated into new cell culture flasks every second week.

### 3D stem-like cell invasion assay

U251MG cells expressing GFP and either NTN1FH or NTN1(II)FH and primary human GBM cells, GBM9 or GBM10, were trypsinized and counted. 1000 U251MG cells and 1000 primary GBM cells were mixed in 100 μl of DMEM/F12 (Gibco) and plated into U-bottom plates (Thermo Scientific) coated with 0,5% agarose. Cells were allowed to form spheroids by incubating them in a cell culture incubator for 24 h. On the following day, six spheroids were collected into Eppendorf tubes and centrifuged gently, 1 min 1000 rpm. The medium was removed and spheroids resuspended into 50 μl of ice-cold Matrigel. The Matrigel and cell mixture was plated into the well of a 24 well plate. The Matrigel droplets were allowed to polymerize at +37 °C 30 min. Next, 1 ml of DMEM/F12 cell culture medium was added to each well. The growth of the spheroids were monitored with Cell-IQ imaging system (Chip Man Tehcnologies) using 10× objective. Both transmitted light and GFP images were acquired. Each spheroid was imaged every 30 min for 24 h. Using Cell-IQ software the growth of the spheroids was analyzed by measuring its area at each imaging timepoint. In addition the images of each spheroids were combined into a movie using 7 frames per second display rate.

### Wholemount immunofluorescence staining of Matrigel drops

The Matrigel droplets were fixed with 4% paraformaldehyde at room temperature for 30 min. Fixed droplets were washed with PBS. The unspecific binding of antibodies was prevented by incubating the droplets with 3% BSA, 0,3% Tween 20 in PBS (blocking buffer) at room temperature for 60 min. Primary antibodies were diluted to blocking buffer, added to droplets and incubated at +4 °C 24 h. Droplets were washed with 0,1% Tween 20 in PBS. Secondary antibodies and Hoechst were diluted to blocking buffer and incubated with the droplets at room temperature for 60 min. The droplets were again washed with 0,1% Tween 20 in PBS. Finally excess salts were removed by washing twice with milli-Q aqua. The droplets were transferred onto microscopic slides, mounted with Mowiol mounting medium supplemented with 6 mg/ml DABCO anti-fading reagent and covered with a cover glass. The spheroids were analyzed and imaged under Zeiss AxioImager.Z2 upright epifluorescence microscope equipped with Hamamatsu Orca Flash 4.0 LT, 4 megapixel monochrome sCMOS camera.

The stemness of the isolated primary cells was ensured by examining their mRNA expression of GBM stem-like cell markers CD133, nestin, Sox2 and integrin alpha 6 with quantitative real time PCR. RNA from the cells was collected and real-time PCR was performed as described earlier [41]. The primers for the analysis were obtained from Applied Biosystems. The expression levels were normalized to the expression of GAPDH and the compared to differentiated GBM10 cells that were cultured in serum containing DMEM/F12 medium and adherent conditions.

### Statistical analysis

Error bars represent the standard deviation or standard error of the mean of the 3–5 independent repeats of each experiment. Statistical significance was analyzed with non-parametric Mann–Whitney *U*-test for independent samples for continuous variables. The frequency tables were analyzed with the *χ* ^2^ test. Cumulative survival was estimated by using the Kaplan–Meyer method. Overall survival for GBMs was calculated from the date of diagnosis to the date of death, censoring patients who were alive on the last follow-up date. Glioma-specific survival was analyzed from the date of surgery of primary tumor to the date of glioma caused death, censoring patients who died from other causes on the date of death and the patients who were alive on the date of last follow-up, and recurrence-free survival was calculated from the date of surgery to the date of recurrence or death if death occurred before recurrence, censoring those patients who died of other causes and the patients who were alive on the date of last follow-up. Multivariable and univariable survival analyses were done by using Cox proportional hazards model.

## Results

### Netrin-1 associates with poor patient survival in low grade gliomas

**Fig. 1:**
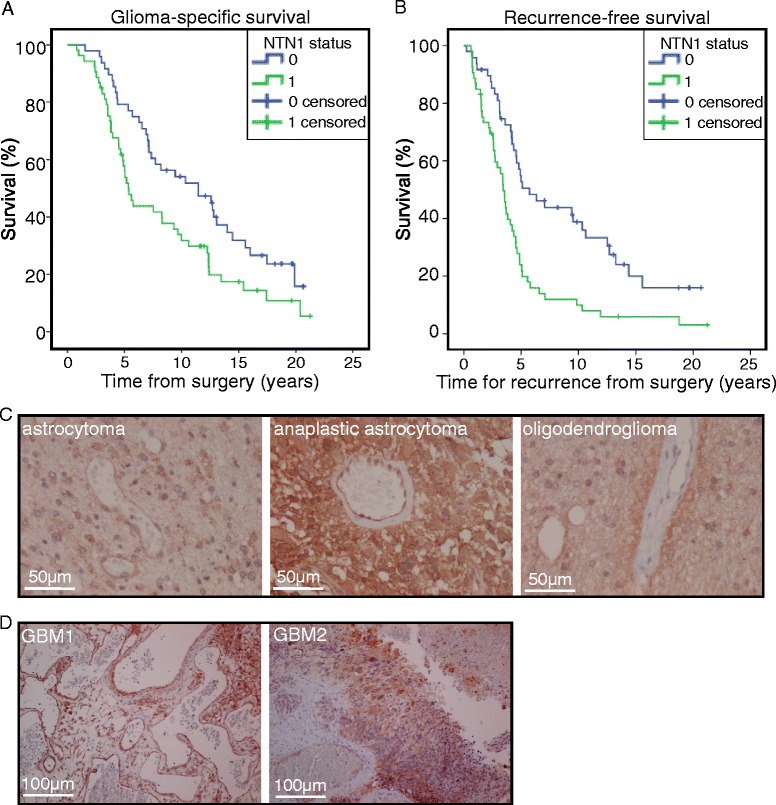
Netrin-1 is associated with poor patient prognosis in gliomas. The association of NTN1 with the survival of glioma patients was assessed using Kaplan-Meier survival analysis. NTN1 association to (**a**) glioma specific survival (hazard ratio [HR] = 1.73, 95% confidence interval [95% CI] = 1.11 to 2.71; *p* = 0.015) and (**b**) to recurrence-free survival were analyzed (HR = 1.62, 95% CI = 1.04 to 2.53; *p* < 0.001). **c** The localization of NTN1 in low grade gliomas was investigated by immunofluorescence staining of paraffin embedded tumor tissue. Scale bars represent 50 μm. **d** The localization of NTN1 in GBM tissue was analyzed similarly as in (**c**). Representative images of two tumors are presented. Scale bars represent 100 μm

NTN1 has been observed to play a role in GBM cell invasiveness and survival in vitro by us and others [33, 41]. However, its association with the survival of glioma patients has not been investigated. To address this we used immunohistochemical stainings and analyzed NTN1 expression in tissue microarrays (TMAs) consisting of 136 representative primary glioma samples [34, 38]. The TMAs consisted of grade II-III astrocytomas, oligodendrogliomas and oligoastrocytomas and grade IV GBMs. NTN1 expression was detected on all tumor subtypes. Within the tissue samples 40.4% of the tumors were NTN1 positive (Table 1). NTN1 was strongly associated with astrocytic tumors (Table 1). Within astrocytomas 77.8%, within anaplastic astrocytomas 62.5% and within GBMs 77.1% of the tumors were NTN1 positive whereas within oligodendrogliomas and oligoastrocytomas only 26.7% and 36.8%, respectively, were NTN1 positive. Furthermore, we observed that NTN1 correlated with nestin expression. Nestin has been connected to the stemness of GBM cells and to poor prognosis of glioma patients [37, 42]. No association with gender or age at the time of diagnosis was found (Table 1). To investigate whether NTN1 expression is related to the survival of the patients we performed Kaplan-Meier survival analysis. Both glioma-specific and recurrence-free survival times were analyzed. Interestingly, NTN1 positivity was associated with poor glioma-specific survival (hazard ratio [HR] = 1.73, 95% confidence interval [95% CI] = 1.11 to 2.71; *p* = 0.015) (Fig. 1a) and with shorter recurrence-free survival time (HR = 1.62, 95% CI = 1.04 to 2.53; *p* < 0.001) (Fig. 1b). However, when astrocytomas and oligodendroglial tumors were analyzed separately NTN1 expression was not significantly associated with patient survival.

**Table 1:** Association between netrin-1 expression and clinicopathological factors

	Netrin-1 expression		
	Negative	Positive	*P* value
Histological type	*n* = 55, % = 40.4	*n* = 81, % = 59.6	
Astrocytoma	8 (22.2)	28 (77.8)	<.0001
Anaplastic astrocytoma	6 (37.5)	10 (62.5)	
Oligodendroglioma	22 (73.3)	8 (26.7)	
Oligoastrocytoma	12 (63.2)	7 (36.8)	
Glioblastoma	8 (22.9)	27 (77.1)	
Gender			
Female	27 (43.5)	35 (56.5)	.499
Male	28 (37.8)	46 (62.2)	
Grade			
II	35 (49.3)	36 (50.7)	.033
III	12 (40.0)	18 (60.0)	
IV	8 (22.9)	27 (77.1)	
Nestin expression			
Negative	14 (51.9)	13 (48.1)	.014
Positive	20 (26.0)	57 (74.0)	
NA	21	11	
Age at the time of diagnosis			
Median/range, years	41 (23–76)	44 (17–75)	.386

*Abbreviations: NA* data not available

Moreover, we investigated the localization of NTN1 in the tumor tissues. In all tumor types NTN1 was localized to hypoxic tumor areas and vasculature including glomerular vessels (Fig. 1c–d). In GBM tissues NTN1 expression was enriched to areas surrounding the necrotic tumor core and especially to pseudopalisade structures (Fig. 1d). The pseudopalisading cell areas are a typical feature of GBMs and contain actively migrating cells [5].

### Netrin-1 colocalizes with Jagged1 but not with Notch2, Nestin or CD133 in GBM tissues

**Fig. 2:**
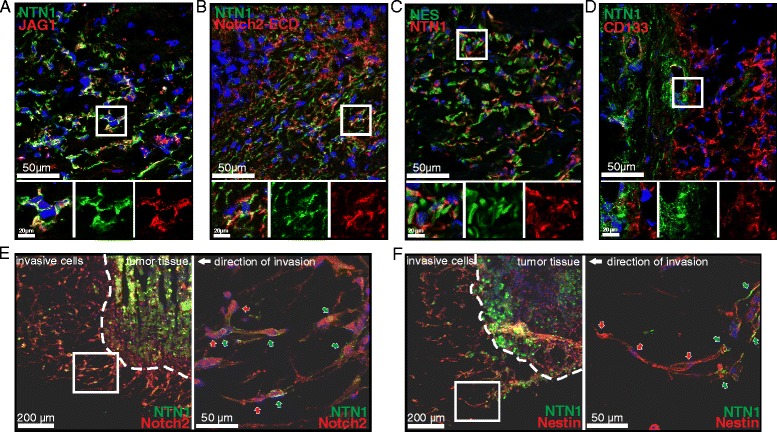
Netrin-1 co-exists with glioblastoma stem like cells in tumor stroma and infiltrative sprouts. **a** Colocalization of NTN1 and Jagged1 was analyzed by immunofluorescence staining of human GBM tissue. Representative area of the full image is surrounded with white box and presented as enlargement in lower panel. Scale bars represent 50 μm in full image and 20 μm in the enlargement. Merged image with all channels and separate channels of the enlargement are presented. NTN1 is marked with *green*, Jagged1 with *red* and nuclei with *blue*. **b** Colocalization of NTN1 and Notch2 was visualized by immunofluorescence staining of human GBM tissue similarly as in (**a**). NTN1 is marked with *green*, Notch2 with *red* and nuclei with *blue*. **c** Colocalization of NTN1 and Nestin was analyzed by immunofluorescence staining of human GBM tissue similarly as described in (**a**). NTN1 is marked with *red*, nestin with *green* and nuclei with *blue*. **c** Colocalization of NTN1 and CD133 was studied by immunofluorescence staining of human GBM tissue similarly as described in (**a**). NTN1 is marked with *green*, CD133 with *red* and nuclei with *blue*. **e** and **f** To study invasive front of human GBM tumor, tissue pieces were grown in 3D Matrigel ex vivo. The location of NTN1 and nestin (**e**) or NTN1 and Notch2 (**f**) was analyzed with immunofluorescence microscopy. The border of tumor tissue and invasive cells is implicated with *dashed line*. Enlargement of the boxed area is presented. In the enlargement *white arrow* points the direction of the migration. *Green arrows* point to NTN1 positive cells and *red arrows* Nestin positive cells in (**e**) and Notch2 positive cells in (**f**)

NTN1 can bind both Jagged1 and Notch2 in vitro [41]. To analyze this in human tumors we stained fresh frozen human GBM tissue biopsies with NTN1 and Jagged1 or Notch2 antibodies. We observed that NTN1 and Jagged1 co-localized on the same cells (Fig. 2a) similarly to in vitro cultured GBM cells [41]. In contrast, NTN1 and Notch2 did not co-localize in same tumor cells. Instead, they were expressed in neighboring cells and co-localized within their cell-cell contacts (Fig. 2b).

Notch signaling has been observed to promote the stemness of the GBM cells [6, 12, 17]. Therefore, we hypothesized that NTN1 affects the glioma stem-like cells. To explore this further we studied NTN1 and its co-localization with known GBM stem-like cell markers nestin and CD133 in GBM tissues. Interestingly, we did not observe co-localization of NTN1 in same cells with either of these markers although positive correlation of nestin and NTN1 expression was found in clinical series. Instead, NTN1 was localized to neighboring cells of nestin expressing cells (Fig. 2c). Similar localization was observed with CD133 (Fig. 2d). In the GBM tissues there were areas with CD133 positive cells surrounded by NTN1 positive cells. These results suggest that NTN1 does not localize to the stem-like cells themselves but to their adjacent cells supporting them in the tissue.

Human surgical GBM biopsies represented primarily the tumor core. The single invasive cells present in the brain tissue cannot be removed in surgical operations. However, these invasive cells are the main reason for the relapses in patients. Therefore we wanted to investigate the localization of NTN1 in these cells too. To mimic the invasive front of GBM we established ex vivo human GBM cultures. We implanted freshly collected GBM biopsies in 3D Matrigel and allowed the cells to grow and migrate for 7 days. The Matrigel plugs were then fresh frozen and sectioned. Immunofluorescence staining of the sections revealed that the front of the invasive structures was positive for Notch2 (Fig. 2e) and for nestin (Fig. 2f) suggesting stemness of the invasive cells. Interestingly, NTN1 positive cells remained at the stalk area of the invasive sprouts.

### Netrin-1 expression enhances glioblastoma invasiveness in vivo

**Fig. 3:**
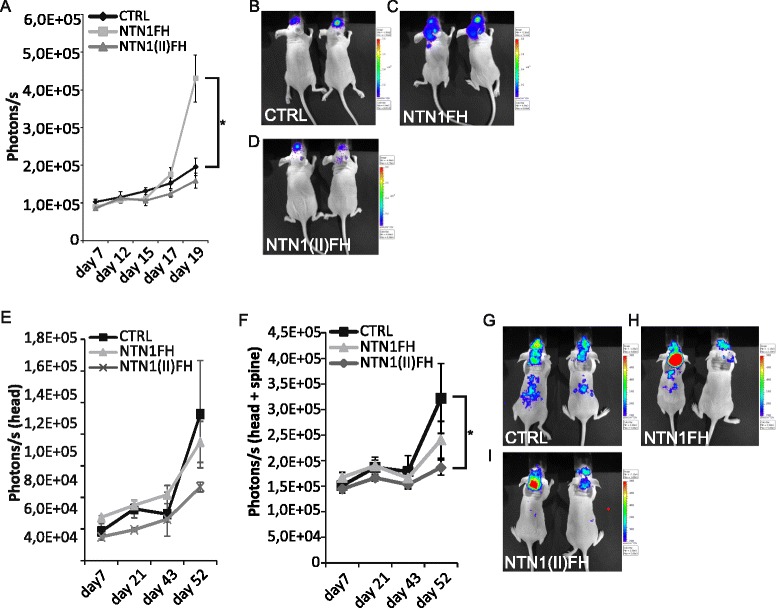
Netrin-1 promotes glioblastoma growth in vivo. **a–d** U87MG or (**e–i**) U373MG cells expressing firefly luciferase and full-length NTN1 (NTN1FH) or its central domain (NTN1(II)FH) were intracranial implanted into nude mice. Five mice per cell-line was xenografted. **a, e** and **f** The growth of the tumors was followed with bioluminescent imaging. Photons emitted by the tumor were recorded and quantitated. The photons emitted by the U373MG xenografts were quantitated separately from the head area of the mice (**e**) and together from the head and spine (**f**). Growth curves represent the average photons/second emitted by the tumors in each group. Error bars represent the standard error of the mean. * *p* value is <0,05. **b-d** and **f-h** Representative image of mice in each xenograft group at the experiment end point are presented. The color bars indicate the intensity of photons emitted by the tumor. The color bar scales are set on equal levels within the groups of U87MG xenografts (**b-d**) and within the groups of U373MG xenografts (**f-h**)

To investigate how NTN1 affects GBM pathogenesis in vivo we performed orthotopic mouse xenografts. We first used U87MG cells because of their low endogenous NTN1 expression. We intracranially implanted either wild type U87MG cells (WT), NTN1 overexpressing U87MG cells (NTN1FH) or cells expressing NTN1 central domain (NTN1(II)FH) into mice (Additional file 1: Figure S1A). NTN1(II)FH peptide can antagonize the effect of NTN1 in in vitro cell invasiveness [41]. The mice were observed for 3 weeks and the tumor growth was estimated based on the luciferase signal emitted by the tumor cells (Fig. 3a). In the first 2 weeks of the experiment there was no difference in the growth of the tumors between the three groups. However, during the third week the NTN1FH tumors started to grow very rapidly. In addition, the NTN1FH tumors had spread all around the head of the mice and in some mice even along their spine (Fig. 3b–c). Interestingly, the growth of the NTN1(II)FH expressing tumors did not significantly differ from the WT tumors (Fig. 3b and d). The number of photons emitted were slightly decreased and the location of tumors restricted to the head of the mice (Fig. 3d). This may be due to the lack of endogenous NTN1 expression in the U87MG cell-line.

To investigate how inhibition of the function of NTN1 affects GBM growth we performed intracranial xenografts using U373MG cell line. These cells endogenously express NTN1 [41] and grow infiltratively in vivo [2, 8]. We implanted either wild type or cells expressing either NTN1FH or NTN1(II)FH peptide (Additional file 1: Figure S1B) into mice brains and followed the tumor growth similarly to U87MG xenografts.

The growth of U373MG tumors was overall much slower than U87MG cells. We followed the mice for 52 days after tumor cell injection. Similarly to U87MG xenografts, tumors in all groups grew similarly to each other in the beginning (Fig. 3e–f). After 6 weeks the groups began to display differences in their growth. Both the control tumors and NTN1FH tumors grew all around the mouse head area and also spread along the spine of the mice whereas the NTN1(II)FH tumors grew exclusively around the head area (Fig. 3g–i). Considering the emitted photons only from the head area of the mice we could not observe any significant differences between the groups (Fig. 3e). However, the signal from both the head and the spine of the mice revealed a significant decrease on the photons within NTN1(II)FH group (Fig. 3f). There was no increase on the invasiveness within the NTN1FH group, possibly due to the endogenous expression of NTN1 in U373MG cells. Taken together these results suggest that NTN1 is an important regulator of GBM growth and invasiveness in vivo and its inhibition prevents the diffusive growth of GBM.

**Fig. 4:**
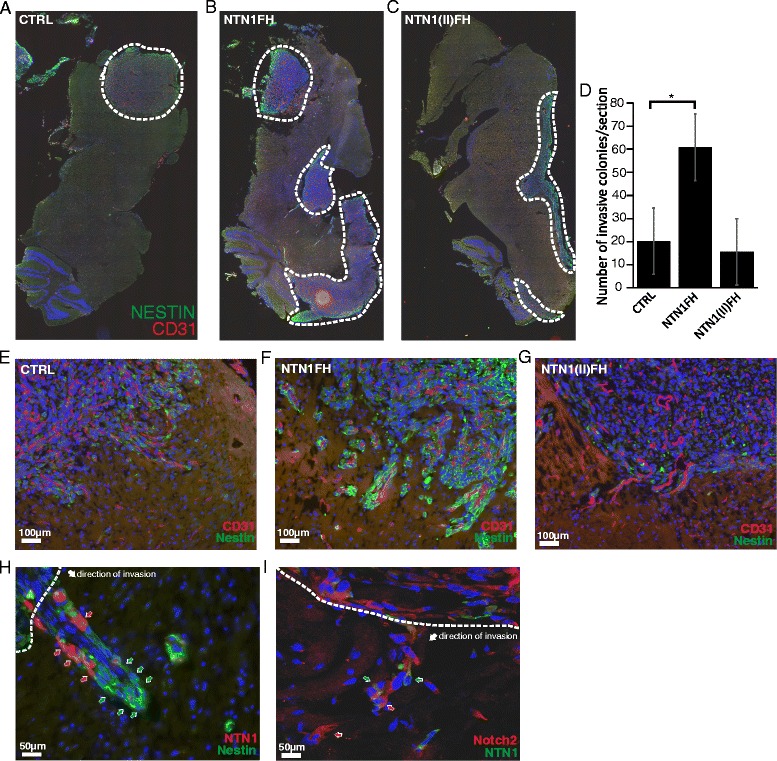
Netrin-1 increases the invasiveness of GBM stem-like cells in vivo. **a–c** The tumor growth in xenograft models was evaluated by immunohistochemistry with human specific nestin antibody. Representative tile images of tissue sections of each U87MG xenograft group are presented. The area covered with tumor is surrounded with *dashed line*. **d** To evaluate the effect of NTN1FH or NTN1(II)FH on GBM cell invasiveness xenograft tissue section were stained with human specific Lamin A/C antibody and the invasive colonies were counted. The average number of invasive colonies in each group is presented. Error bars represent +/− standard error of the mean. * *p* value is <0,05. **e–g** Immunofluorescence microscopy images of the tumor border show the infiltrative growth of nestin positive GBM cells in each U87MG xenograft groups. Scale bars represent 100 μm. **h–i** The invasive tumor sprouts of U87MG-NTN1FH tumors were further characterized with immunofluorescence stainings of the tissue sections. *Dashed line* borders the tumor stroma and the *white arrow* points the direction of invasion. In (**h**) *red arrows* point NTN1 positive cells and *green arrows* Nestin positive cells. In (**i**) *green arrows* point NTN1 positive cells and *red arrows* the Notch2 positive cells. Scale bars represent 50 μm

After sacrificing the mice, we harvested their brains and analyzed the growth and invasiveness of the tumors by staining tissue sections. First, the expression of NTN1FH and NTN1(II)FH both in U87MG and U373MG xenograft tumors was confirmed (Additional file 1: Figure S1C and D). We next analyzed whether the differences observed in the xenograft tumor growth were due to altered cell proliferation. The proliferation rates of cultured cells used for xenografts were measured using EdU incorporation assay. We observed slight increase of U87MG cell proliferation upon NTN1FH expression (Additional file 1: Figure S1E). However, we did not observe similar effect in U373MG cells (Additional file 1: Figure S1D). Consistently with luminescence measurements, the control and NTN1(II)FH U87MG cells had formed a solid tumor with few invasive structures. Furthermore, NTN1 expression increased tumor growth and altered the growth into more invasive phenotype (Fig. 4a–c). The cells were spreading along the blood vessels adjacent to the tumors (Fig. 4b). To quantify the invasiveness of the tumor cells we stained the tissue sections with human specific lamin A/C antibody which enabled us to distinguish the human cells from the mouse tissue. The invasive cells were then counted in a double blinded manner. Significant increase in the number of invasive colonies was observed in the NTN1FH U87MG tumors (Fig. 4d) and consistently lower number in the NTN1(II)FH U373MG tumors (Additional file 2: Figure S2).

Because NTN1 expression correlated with nestin positivity in human glioma TMA we analyzed nestin expression in the GBM xenografts. Unexpectedly, we detected that the invasive edge of the U87MG NTN1FH tumors was strongly nestin positive in NTN1FH tumors but not in control or NTN1(II)FH tumors (Fig. 4e–g). Furthermore, the border of tumor stroma and the normal mouse brain tissue was clear-cut in the control and NTN1(II)FH tumors but more diffuse in NTN1FH tumors. We further stained the NTN1FH tumor sections for nestin and HA to localize NTN1 positive cells in the tumors. We observed that the invasive structures consistently showed an assembly where the nestin positive cells were on the leading edge whereas NTN1 positive cells were following them (Fig. 4h). We also observed similar pattern with Notch2 staining (Fig. 4i). These findings are consistent with the observations in the human ex vivo GBM models where the leading cells of the invasive structures were nestin and Notch positive whereas NTN1 positive cells were in the stalk area of the structures (Fig. 2e–f).

### Netrin-1 increases the percentage of GBM stem-like cells and enhances their motility

**Fig. 5:**
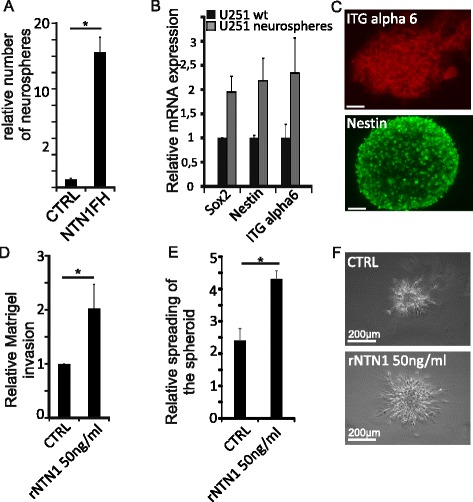
Netrin-1 promotes the maintenance and motility of U251MG stem-like cells. **a** Either wild-type or NTN1 overexpressing U251MG cells were cultured under condition that favor neural stem cell proliferation for 3 weeks. The formed neurospheres were calculated. The number of the neurospheres is presented as relative to the wildtype U251MG. Error bars represent SD. * = *p* < 0,05. **b** The formed U251MG neurospheres were collected and the expression of stem cell markers was evaluated with quantitative real time PCR. The mRNA expression is normalized to the expression of GAPDH. The results are expressed as relative to the mRNA expression levels of U251MG cells cultured under standard conditions. Error bars represent the standard deviation. **c** The expression of the markers was also confirmed with immunofluorescence microscopy. Scale bars represent 50 μm. **d** U251MG neurospheres were dissociated and plated into Matrigel coated Boyden chambers. Cells were allowed to invade for 8 h and then quantitated. Number of invaded cells are presented as relative to the control group. Error bars represent SEM and * = *p* < 0,05. **e** Neurospheres were embedded into 3D Matrigel matrix and monitored for 24 h. The area of the spheroids was measured at the starting and ending point of the experiment. The graphs represent the relative change in the area of the spheroid. Error bars represent SEM and * = *p* < 0,05. **f** Images represent the endpoint of the 3D Matrigel invasion assays. The invasion of the control spheroids is presented also on Additional file 4: Video 1 and the invasion of the rNTN1 treated spheroids in Additional file 5: Video2. Scale bars represent 200 μm

To explore how NTN1 affects the GBM stem-like cells, we cultured both wild type and NTN1 overexpressing U251MG GBM cells under conditions that favor neural stem cell proliferation. Under these culture conditions stem-like cell proliferation leads to the formation of a spheroid called neurosphere [40]. The number of neurospheres formed was calculated and used as an estimate of the number of stem-like cells within the initial population (Fig. 5a). Interestingly, NTN1 overexpression led to 16-fold higher neurosphere formation compared to wild-type cells. These neurospheres also expressed more stemness markers Sox2, nestin and integrin alpha6 compared to normal U251MG cells (Fig. 5b–c). This suggests that the initial NTN1 overexpressing cell population contained a higher number of stem-like cells and that NTN1 plays a role in their maintenance.

Next, we investigated how NTN1 affects the motility of the stem-like cells. We dissociated the U251MG neurospheres and plated the cells on top of Matrigel coated Boyden chamber. After 8 h the cells that had migrated through the chamber were quantified. Addition of 50 ng/ml of recombinant NTN1 (rNTN1) as attractant to the lower chamber significantly increased the number of invading cells (Fig. 5d). To better mimic the 3D tissue environment, we implanted the U251MG neurospheres in 3D Matrigel and monitored their growth for 24 h. Similarly to the Boyden chamber assay, addition of rNTN1 to the medium enhanced the motility of the cells: the spheroids spread much faster upon rNTN1 addition (Fig. 5e). Furthermore, the control spheroids remained more compact compared to rNTN1 treated (Fig. 5f, Additional file 4 and Additional file 5). These results further confirm that rNTN1 regulates the motility of GBM stem-like cells.


Additional file 4: Invasion of U251MG neurospheres in 3D Matrigel. Video (file format QuickTime, extension.mov) represents the invasion of U251MG neurospheres that are embedded into 3D Matrigel. Images were captured with Cell IQ imaging system using 10x objective. Frames were collected every 30 min during 24 h. Display rate is 7 frames/second. Video is related to Fig. 5f.
Additional file 5: Invasion of rNTN1 treated U251MG neurospheres in 3D Matrigel. Video (file format QuickTime, extension.mov) represents the invasion of U251MG neurospheres that are embedded into 3D Matrigel and treated with recombinant NTN1. Images and video were captured similarly as in Additional file 4. Video is related to Fig. 5f.


Freshly isolated GBM cell-lines that are kept under the stem-like cell proliferation favoring culture conditions are a better model for investigating glioma stem-like cells than long cultured cell lines [1, 40]. Therefore we established primary GBM stem-like cell cultures (GBM9 and GBM10) from surgical GBM biopsies. The expression of GBM stem-like cell markers CD133, nestin and Sox2 in stem-like cell cultures was higher compared to counterpart cells cultured in serum containing medium, confirming their stemness (Additional file 3: Figure S3A).

GBM tumors are heterogeneous and contain both stem-like cells and differentiated tumor cells. To mimic this we dispersed stem-like GBM9 or GBM10 spheroids and mixed these cells with GFP labelled U251MG cells cultured in serum containing medium. Furthermore, we had observed that NTN1 induces invasion mode where nestin positive cells were enriched at the invasive front of the tumors and NTN1 positive cells were following them (Figs. 2e–f and 4e–i). To explore this we expressed NTN1FH or NTN1(II)FH peptides in the U251MG cells and mixed them with GBM9 or GBM10 cells. The mixed cell populations were allowed to form spheroids under serum free conditions and embedded into 3D Matrigel matrix and observed for 24 h.

**Fig. 6:**
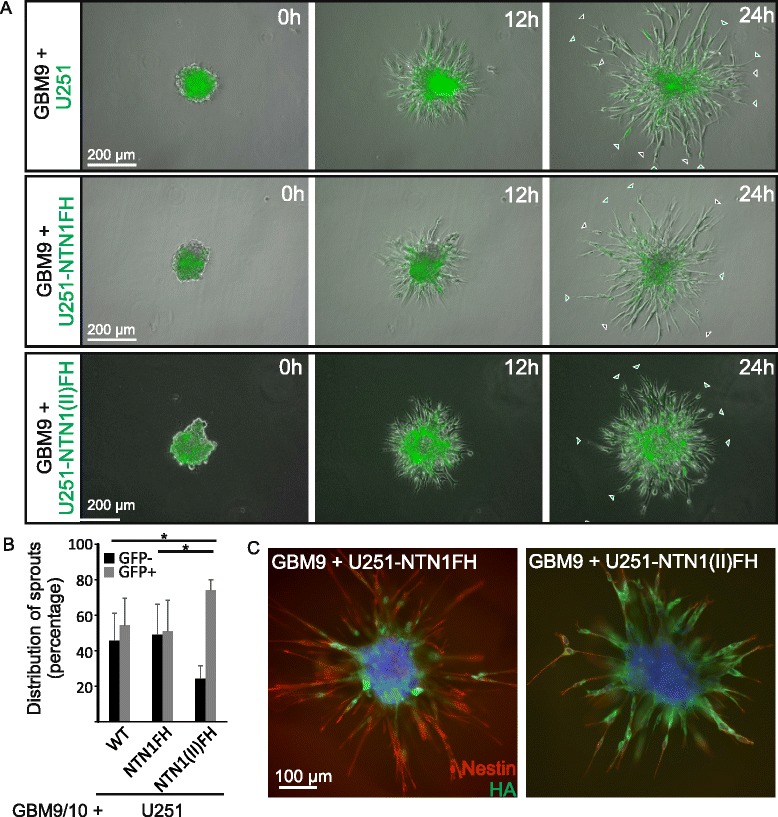
Netrin-1 promotes the motility of the primary glioblastoma stem-like cells. **a** Primary human GBM stem-like cells, GBM9, were mixed with GFP positive U251MG cells, that were either wildtype (U251), full length NTN1 producing (U251-NTN1FH) or NTN1 central domain producing (NTN1(II)FH). The spheroids were implanted into 3D Matrigel and their growth was monitored with Cell-IQ imaging system for 24 h. *Gray arrows* mark sprouts that are led by GBM9 cells. *Green arrows* mark sprouts that are led by GFP-positive U251MG cells. The invasion of the spheroids is presented also on Additional file 6: Video3, Additional file 7: Video4 and Additional file 8: Video5. **b** The number of sprouts led by the GFP-negative (GFP-) primary cells or GFP-positive (GFP+) U251MG cells were quantitated. The distribution as percentage is presented. Error bars represent SD. * = *p* < 0,05. **c** After 24 h 3D Matrigel invasion spheroids consisting of GBM9 cells mixed with U251-NTN1FH or U251-NTN1(II)FH were fixed and used for immunofluorescence staining against Nestin and HA. HA marks either full-length NTN1 (NTN1FH) or the central fragment of NTN1 (NTN1(II)FH)


The spheroids composed of GBM9 and control or NTN1FH expressing U251MG invaded diffusively into the Matrigel (Fig. 6a, Additional file 6 and Additional file 7). There were two types of sprouts invading: sprouts where the GFP positive U251MG cells were leading and sprouts where GBM9 cells were leading. In contrast, the spheroids composed of GBM9 and U251-NTN1(II)FH cells invaded in more compact manner and presented only GFP positive sprouts (Fig. 6a, Additional file 8). To quantify the ratio of sprouts we fixed the invaded spheroids and counted the percentage of GBM stem-like cell or U251MG guided sprouts in the different spheroid types (Fig. 6b). In GBM9/10-U251 and in GBM9/10-U251-NTN1FH spheroids both sprout types existed at about equal proportions. In GBM9/U251MG-NTN1(II)FH spheroids more than 80% of the sprouts were led by U251GM cells (Fig. 6b).

Additional file 6: Invasion of GBM9/U251MG mixture spheroids in 3D Matrigel. Video (file format QuickTime, extension.mov) represents the invasion of spheroids consisting of GBM9 and U251MG-plvx cells. The spheroids were embedded into 3D Matrigel and monitored for 24 h. Transmitted light and epifluorescence images were captured with Cell IQ imaging system using 10x objective. Frames were collected every 30 min. Display rate is 7 frames/second. Frames of various time points are presented in Fig. 6b.
Additional file 7: Invasion of GBM9/U251MG-NTN1FH mixture spheroids in 3D Matrigel. Video (file format QuickTime, extension.mov) represents the invasion of spheroids consisting of GBM9 and U251MG-NTN1FH cells. The spheroids were embedded into 3D Matrigel and monitored for 24 h. Images and video were captured similarly as in Additional file 6. Frames of various time points are presented in Fig. 6b.
Additional file 8: Invasion of GBM9/U251MG-NTN1(II)FH mixture spheroids in 3D Matrigel. Video (file format QuickTime, extension.mov) represents the invasion of spheroids consisting of GBM9 and U251MG-NTN1(II)FH cells. The spheroids were embedded into 3D Matrigel and monitored for 24 h. Images and video were captured similarly as in Additional file 6. Frames of various time points are presented in Fig. 6b.


We also analyzed the differences in the overall invasiveness of the different mixed spheroids by measuring the area of the spheroid in various time points. We observed that NTN1FH expression slightly increased the spheroid area while the NTN1(II)FH decreased it (Additional file 3: Figure S3B). These observations are in line with the differences in the xenograft growth.

**Fig. 7:**
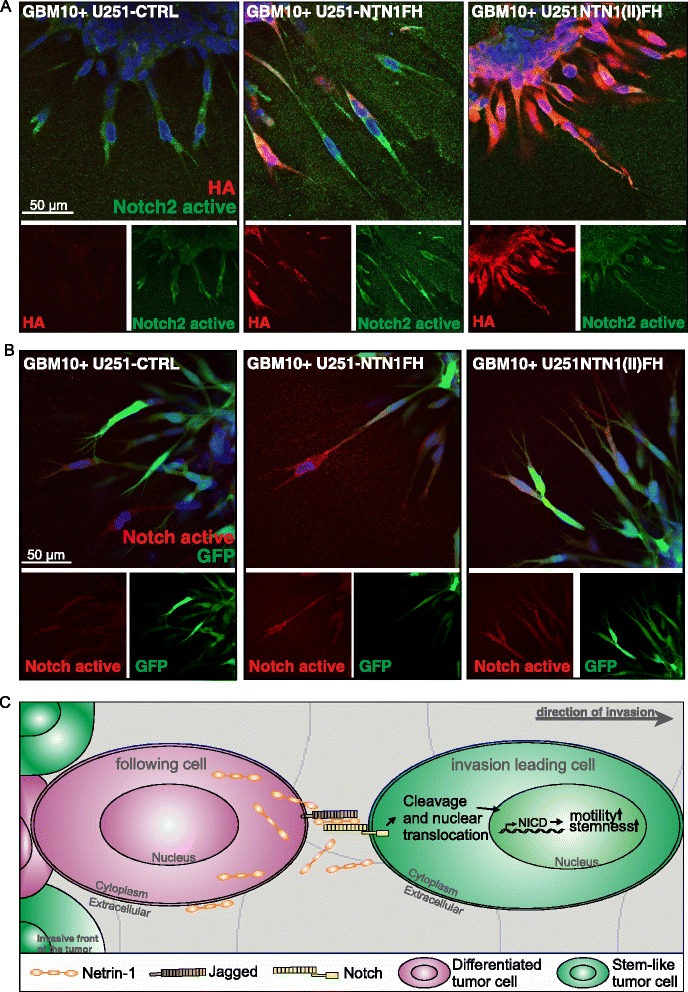
Invasive primary GBM cells increase Notch2 activation upon NTN1FH expression. **a** After 24 h 3D Matrigel invasion spheroids consisting of GBM10 cells mixed with control U251, U251-NTN1FH or U251-NTN1(II)FH were fixed and used for immunofluorescence staining against cleaved Notch2 and HA. HA marks either full-length NTN1 (NTN1FH) or the central fragment of NTN1 (NTN1(II)FH). **b** Similarly as in (**a**) the invaded spheroids consisting of GM10 cells mixed with GFP positive U251MG cells were stained against cleaved Notch2. **c** Model of NTN1 induced invasion of GBM cells

As a portion of cultured U251MG cells have stem-like properties and nestin expression, the co-culture spheroids were also stained for nestin. In the GBM9/U251-NTN1FH spheroids practically all of the sprouts were nestin positive whereas the HA positive NTN1 expressing cells were mostly either in the core of the spheroids or in the stalk area of the sprouts (Fig. 6c). In contrast, in the GBM9/U251-NTN1(II)FH spheroids the majority of the sprouts were HA positive indicating that they are of U251MG origin. Even in these spheroids the tips of the invading cells were nestin positive. Furthermore, we investigated whether the sprouts exhibited differences in Notch activation. Interestingly, we observed that in NTN1FH spheroids the sprouts led by GFP and HA negative cells were positive for Notch cleavage which indicates the signaling activation (Fig. 7a and b). Similarly in spheroids containing control U251 cells Notch positive GFP negative sprouts were observed. In contrast, in the presence of U251-NTN1(II)FH the staining for active Notch was less intense. In these spheroids all of the invasive sprouts were GFP and HA positive, indicating that the invasive cells were of U251 origin and expressed NTN1(II)FH (Fig. 7a and b). Taken together these results suggest that NTN1 regulates the GBM invasiveness by promoting the motility of the GBM stem-like cells and the inhibition of the NTN1 signaling with NTN1(II)FH peptide reduces the invasiveness of especially GBM stem-like cells.

### Discussion

GBM is the most severe human brain cancer. Despite numerous efforts within recent years, no curative treatments have been discovered. In addition to the difficult location, GBM tumors are very heterogeneous and spread aggressively. Their infiltrative growth type hinders the surgical removal of all tumor cells. Because GBM cells are enormously plastic and can resemble characteristics of stem cells the remaining cells rapidly grow into secondary tumors [3, 4]. In the current research we provide a novel insight into the regulation of invasiveness of GBM using in vivo and in vitro models.

NTN1 is a secreted extracellular matrix component. In tissue microarray analysis of gliomas NTN1 was strongly linked to poor patient prognosis and especially to tumors of astrocytic origin. This subtype of gliomas are known to be more invasive than the oligodendrocytic tumors [26]. Furthermore, NTN1 located on hypoxic tumor areas that contain motile GBM stem-like cells. In orthotopic xenograft models, the expression of NTN1 increased the total tumor mass. Netrin-1 overexpression caused a subtle increase in tumor cell proliferation, which may explain this increase to some extent. However, netrin-1 was able to alter the behavior of non-invasive U87MG GBM cell-line into more diffusively invading. Similarly, the inhibition of NTN1 signaling by a recombinant NTN1(II)FH peptide could reverse the phenotype of diffusively invasive U373MG cells into less invasive. Taken together, these results describe NTN1 as a powerful regulator of glioma invasiveness.

During developmental processes NTN1 is linked to the regulation of cell motility and stem cell self-renewal. It increases the motility of blood derived mesenchymal stem cells via matrix metalloprotease dependent breakdown of E-cadherin [19]. In addition, NTN1 increases the self-renewal of embryonic stem cells by preventing classical NTN1 receptor, UNC5B, dependent apoptosis [25]. During the lung bud development, NTN1 is accumulated to the stalk area of the developing lung bud but not the growing tip of the branch [21]. Similarly, in the developing mammary gland, NTN1 is expressed by the pre-luminal cells that are located behind the migrating cap cells [7]. Here, we observed similar NTN1 mediated regulation of cancer stem-like cell motility and self-renewal. We observed that in the NTN1 expressing U87MG xenografts tumors the boundary of the tumor and the normal brain tissue was positive for stemness indicating marker nestin. In the control tumors or those expressing NTN1(II)FH peptide this was not observed. Interestingly, when we examined the invasive colonies or sprouts that were growing away from the tumor core, we observed a distinct assembly. The cells in the leading edge were positive for nestin whereas the cells in the stalk area of the sprouts were NTN1 positive. Similar structures were visible in both xenograft models and in ex vivo human GBM tissue cultures. The structure of these sprouts was similar to those observed in NTN1 guided lung and mammary gland morphogenesis [7, 21]. These findings support the role of NTN1 as regulator of stem cell motility.

In our previous work we found that NTN1 can upregulate Notch2 signaling in human GBM cells in vitro [41]. In addition, elevated Notch signaling has been connected to the maintenance of the stem-like cells in GBM tumors [6, 12, 17] and to the upregulation of nestin expression [32]. Therefore we speculated that the NTN1 induced upregulation of invasiveness and stem-like phenotype of GBM cells is mediated by Notch signaling. Indeed, we observed that when NTN1 was overexpressed in GBM cells in vitro their percentage of stem-like cells was increased. This suggests that NTN1 promotes the maintenance of the stem-like cells within the population. In the human GBM tissue NTN1 colocalized with a known Notch ligand Jagged1. On the contrary, Notch2 and NTN1 were not expressed in same cells but they did colocalize on the cell-cell contacts of neighboring cells. Similar coexpression in neighboring cells was observed with NTN1 and GBM stemness markers CD133 and nestin. Furthermore, the nestin positive leading edges of the invasive colonies in both the xenograft tumors and in the ex vivo cultured GBM tumors were also positive for Notch2. Moreover, NTN1 inhibition in the co-culture of human primary GBM cells and U251MG cells significantly decreased the ratio of invasive, Notch activated primary stem-like cells. Taken together, these findings provide a new mechanism for GBM invasiveness. NTN1 in the stalk area of the invasive structures can activate the Notch signaling in the adjacent cells that form the leading edge of the sprouts (Fig. 7c). This allows the maintenance of stem like characteristics of the leading cells which is a great advantage because the stem like cells are very plastic and motile.

Infiltrative invasiveness and the stemness of the tumor cells are hallmarks of GBM and also the main reasons for the treatment failure [23]. The current treatment regimens offer only modest prolongation of the survival but do not cure the disease [39]. Therefore there is a need for new treatment options. Here we describe NTN1 as a novel regulator of GBM invasiveness and stemness. By inhibiting NTN1 signaling both of these phenotypes can be targeted. Therefore, NTN1 signaling inhibition could offer a powerful way to target GBM. Furthermore, we have previously engineered a NTN1(II)FH peptide that can overcome the effects of the full-length NTN1 [41]. Here we provide evidence that this peptide can inhibit GBM growth in vivo by specifically targeting the stem-like cells. This peptide may be of therapeutic value for GBM treatment.

## Conclusions

This study demonstrates that NTN1 is an important regulator of stemness and motility of glioblastoma cells. Although previous studies have implicated that NTN1 may regulate cancer cell invasion this is the first time a connection to cancer stem cells is described. Furthermore, it deciphers a novel mechanism where NTN1 activates Notch signaling and subsequent stemness in invasive glioblastoma cells.

## Additional files

Additional file 1: Figure S1. NTN1FH and NTN1(II)FH are expressed in U87MG and U373MG xenografts. (A) U87MG cells stably expressing NTN1FH and NTN1(II)FH were lysed and immunoblotted against HA. Positions of known protein markers are presented. (B) Expression of NTN1FH and NTN1(II)FH in U373MG cells was similarly validated as in (A). (C) and (D)NTN1FH and NTN1(II)FH expression was confirmed in U87MG xenograft tumors (C) and U373MG xenograft tumors (D) with immunofluorescence staining against HA-tag. (E) and (F) The effect of NTN1FH and NTN1(II)FH to cell proliferation was analysed using EdU incorporation assay and automated image analysis. Effect to U87MG cells (E) and to U373MG cells was analyzed. (PDF 1832 kb)
Additional file 2: Figure S2. NTN1(II)FH alters U3737MG invasiveness in vivo. The number of invasive colonies in U373MG xenografts was analyzed similarly as in Fig. 4D. (PDF 1351 kb)
Additional file 3: Figure S3. Primary human GBM cells express stem cell markers. (A) The stemness of primary GBM cells was assessed by quantitative real-time PCR. The expression was normalized to GAPDH expression. Error bars represent the standard error of the mean. (B) Primary stem-like GBM cells were mixed with U251 cells and embedded into Matrigel. The spheroids were allowed to invade for 24 h. The spheroids were imaged every 15 min. The area covered by the spheroids were measured in multiple time points. The growth curves represent the change of the area of spheroids. Error bars represent SEM. (PDF 1365 kb)

## Abbreviations

bFGF: basic fibroblast growth factor
DMEM: Dulbecco’s modified essential culture medium
EGF: Epidermal growth factor
GAPDH: Glyceraldehyde 3-phosphate dehydrogenase
GBM: Glioblastoma multiforme
HA: Hemagglutinin
NTN1(II)FH: Central domain of netrin-1 fused with FLAG and HA tags
NTN1: Netrin-1
NTN1FH: Netrin-1 fused with Flag and HA tags
PBS: Phosphate buffered saline
rNTN1: recombinant netrin-1
TBS: Tris buffered saline
