# Supplementary figures and images for: Motility of glioblastoma cells is driven by netrin-1 induced gain of stemness
Source: J Exp Clin Cancer Res. 2017 Jan 9;36:9. doi: 10.1186/s13046-016-0482-0 (PMC5223529; doi:10.1186/s13046-016-0482-0)

Figure S1

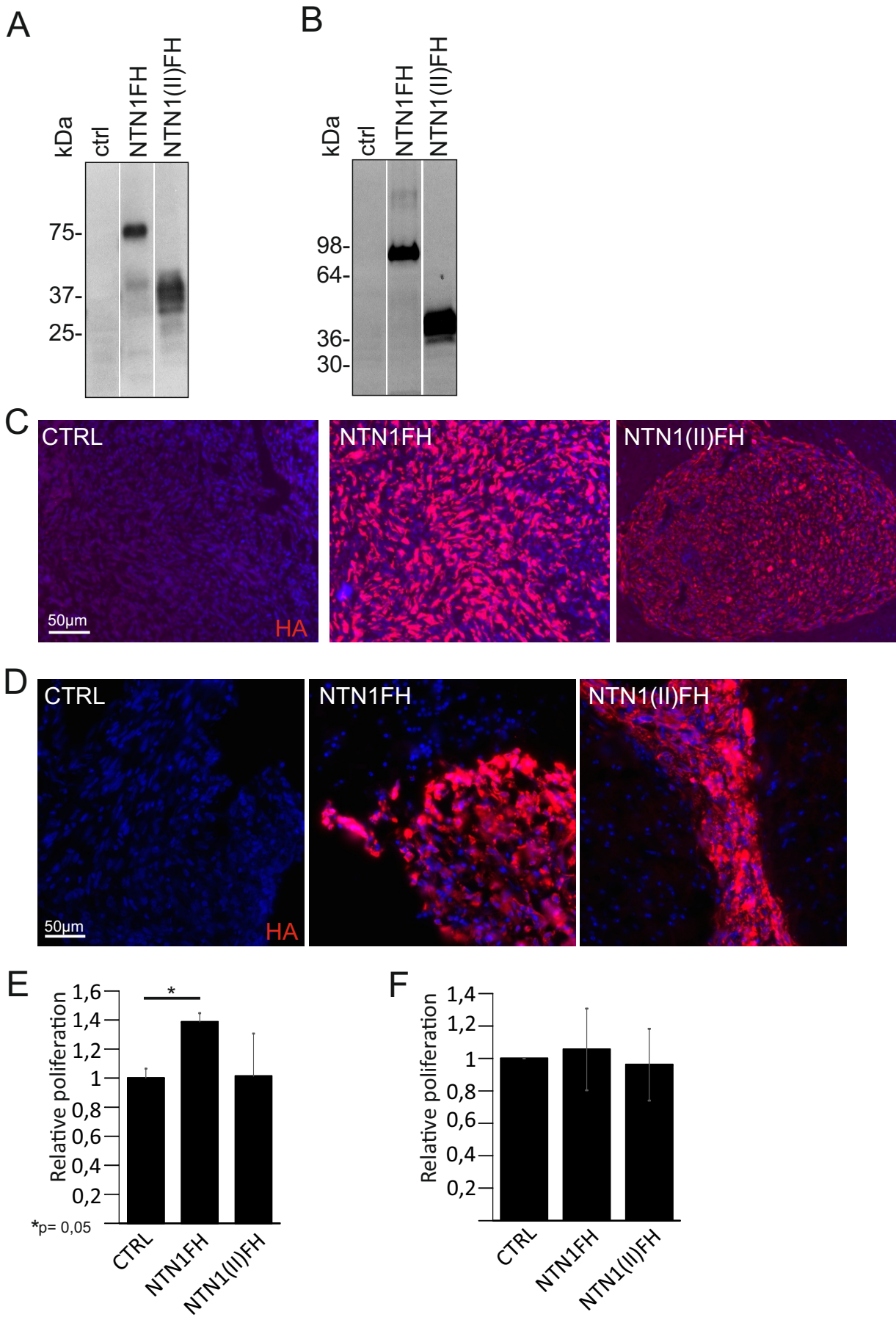

Supplement: Additional file 1: Figure S1. — NTN1FH and NTN1(II)FH are expressed in U87MG and U373MG xenografts. (A) U87MG cells stably expressing NTN1FH and NTN1(II)FH were lysed and immunoblotted against HA. Positions of known protein markers are presented. (B) Expression of NTN1FH and NTN1(II)FH in U373MG cells was similarly validated as in (A). (C) and (D)NTN1FH and NTN1(II)FH expression was confirmed in U87MG xenograft tumors (C) and U373MG xenograft tumors (D) with immunofluorescence staining against HA-tag. (E) and (F) The effect of NTN1FH and NTN1(II)FH to cell proliferation was analysed using EdU incorporation assay and automated image analysis. Effect to U87MG cells (E) and to U373MG cells was analyzed. (PDF 1832 kb) [file 13046_2016_482_MOESM1_ESM.pdf]

Figure S2

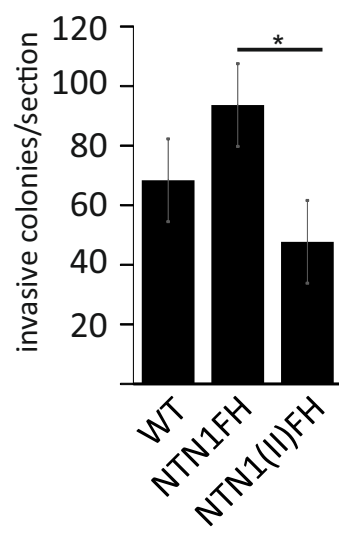

Supplement: Additional file 2: Figure S2. — NTN1(II)FH alters U3737MG invasiveness in vivo. The number of invasive colonies in U373MG xenografts was analyzed similarly as in Fig. 4D. (PDF 1351 kb) [file 13046_2016_482_MOESM2_ESM.pdf]

Figure S3

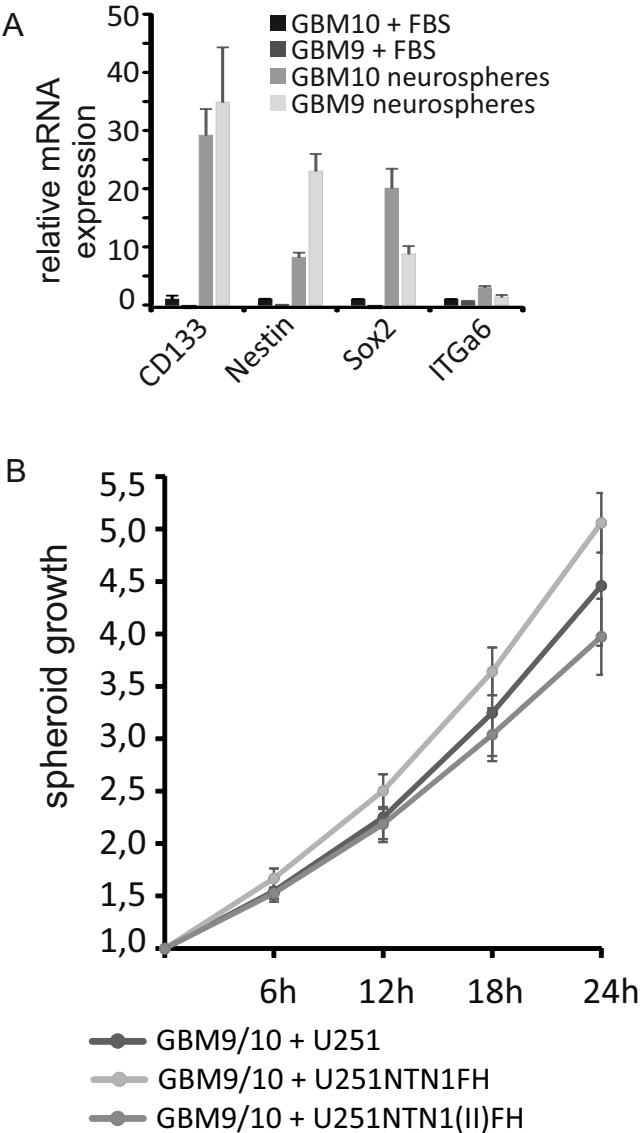

Supplement: Additional file 3: Figure S3. — Primary human GBM cells express stem cell markers. (A) The stemness of primary GBM cells was assessed by quantitative real-time PCR. The expression was normalized to GAPDH expression. Error bars represent the standard error of the mean. (B) Primary stem-like GBM cells were mixed with U251 cells and embedded into Matrigel. The spheroids were allowed to invade for 24 h. The spheroids were imaged every 15 min. The area covered by the spheroids were measured in multiple time points. The growth curves represent the change of the area of spheroids. Error bars represent SEM. (PDF 1365 kb) [file 13046_2016_482_MOESM3_ESM.pdf]
